# Characterization of garlic endophytes isolated from the black garlic processing

**DOI:** 10.1002/mbo3.547

**Published:** 2017-10-09

**Authors:** Zhichang Qiu, Xiaoming Lu, Ningyang Li, Mingjie Zhang, Xuguang Qiao

**Affiliations:** ^1^ College of Food Science and Engineering Shandong Agricultural University Tai'an China

**Keywords:** black garlic, endophytes, isolation, identification, *Bacillus*

## Abstract

The objectives of this study were to isolate and identify garlic endophytes, and explore the characteristics of dominant strains. Garlic endophytes were studied through phenotypical characterization and comparative sequence analysis of 16S rDNA based on culture‐dependent approaches. Representative strains inferred from 16S rDNA sequencing were selected for further identification by *gyrA* and *rpoB* gene loci and phylogenetic analysis based on concateneted house‐keeping sequences. Seven kinds of *Bacillus* were found from garlic and black garlic, respectively. Further studies demonstrated that the total bacteria and endophytes showed a sharp decrease firstly, followed by a rapid rise, then maintained at a certain level, and finally slowed down during the black garlic processing. *B. subtilis*,* B. methylotrophicus,* and *B*. *amyloliquefaciens* were the dominant strains. The selected strains were capable of fermenting glucose, lactose, sucrose, and garlic polysaccharide to produce acid but no gas, with a strong ability of heat resistance. The results indicated that there were a certain number of garlic endophytes during the black garlic processing, and *Bacillus* was the dominant strains under the conventional culture‐dependent methods. This report provided useful information for the presence and type of garlic endophytes during the black garlic processing, which were of great significance to study the formation mechanism and quality improvement of black garlic in the future, as well as the security of garlic powder.

## 1 INTRODUCTION

Endophytes can be defined as those microbes that colonize the internal tissues of healthy plants, showing no obvious external sign of infection or negative effect on their host. There have been a hundred years of history on the research of endophytes, with endophytes found in almost every plant studied (Ryan, Germaine, Franks, Ryan, & Dowling, 2008). Plant endophytes which coexist with host plants for a long term can produce a series of the same bioactive secondary metabolites as the host plants, such as antitumor bioactive substances, with great potential for medical, agricultural, and industrial exploitation (Kim et al., 2007).

Garlic (*Allium sativum* L), a member of the family Alliaceae, enjoys the reputation of “antibiotics grown out of the land” (Raghu, Lu, & Sheen, 2012). Rahman (2007) reported that fructose‐containing carbohydrates were the main component of dry garlic, followed by sulfur compounds, proteins, fibers, and free amino acids. Garlic has a wide range of purposes on account of high nutritional value and unique flavor, regarded as one of the daily best healthy food, as demonstrated by some researchers (Ban et al., 2009; Benkeblia, 2004; Kim, 2016; Raghu et al., 2012). Black garlic, a novel garlic deep‐processed product, is obtained by maintaining fresh garlic at high temperature and controlled humidity for a period of time without any additional additives (Bae, Cho, Won, Lee, & Park, 2014; Toledano‐Medina, Pérez‐Aparicio, Moreno‐Rojas, & Merinas‐Amo, 2016). After processing, the functional components of black garlic increased significantly, such as reducing sugar, polyphenols, organic acids, and β‐carboline alkaloids, giving a more powerful efficacy than garlic (Lu, Li, Qiao, Qiu, & Liu, 2016). There are many reports about the bioactivity of black garlic on health, such as antioxidation (Lee et al., 2009), antiaging (Lee & Kim, 2010), hypoglycemic activity (Seo, Gweon, Lee, Kang, & Kim, 2009), antitumor (Dong et al., 2014), immunity enhancement (Wang et al., 2010), etc.

A few researchers declare that black garlic is a type of fermented products made by spontaneous fermentation of whole garlic bulbs (Kim et al., 2012; Lee et al., 2011; Sato, Kohno, & Niwano, 2006). However, little was known about existence and role played by microorganisms during the black garlic processing. Several studies had shown that there were a certain number of endophytes in garlic, which were mainly identified as bacteria and fungi. Shentu, Zhan, Ma, Yu, and Zhang (2014) had isolated an endophytic fungus strain 0248 from garlic, identified as *Trichoderma brevicompactum* based on morphological characteristics and the nucleotide sequences of ITS1‐5.8S‐ITS2 and *tef1*, with a marked inhibitory activity on *Rhizoctonia solani* and *Botrytis cinerea* due to trichodermin. The separation of endophytes from garlic and its bacteriostatic effect were conducted by Wei, Liu, Li, and Zhou (2013), indicating that endophytic bacteria occupied the majority of strains isolated from garlic, with a strong ability of acid production. Nevertheless, there are few reports on the changes in microorganisms during the black garlic processing, occurred high temperature and high humidity.

In general, 16S rDNA gene, which is highly conserved throughout prokaryotic organisms, is regarded as a framework for modern bacterial classification, but it has often proved to be insufficient and show limited variation for the discrimination of closely related taxa (Chun & Bae, 2000). Protein‐coding genes with higher mutation rates have been used for the differentiation and identification of closely related taxa in supplement to 16S rDNA. The *gyrA* gene (coding for DNA gyrase subunit A) and *rpoB* gene (encoding the RNA polymerase β‐subunit), have been used as markers for bacterial accurate identification and for phylogenetic study of *B. subtilis* and related taxa, as demonstrated by some researchers (Chun & Bae, 2000; Palmisano, Nakamura, Duncan, Istock, & Cohan, 2001).

In this study, the endophytes in garlic and black garlic samples were separated, characterized and identified based on conventional morphological approaches and molecular biological approaches. 16S rDNA sequencing was used for the first identification of isolates. Representatives of the different types based on 16S rDNA sequencing were selected for further identification by *gyrA* and *rpoB* sequencing and phylogenetic analysis based on these concateneted house‐keeping sequences. Then properties of dominant endophytes were explored to discover the strains used for the black garlic processing. The results provided a insight into the presence and type of garlic endophytes, which contributed to the further research of black garlic formation mechanism and quality improvement, and clarified the source of *Bacillus* in garlic powder for the security.

## 2 MATERIALS AND METHODS

### 2.1 Samples

Garlic (*Allium sativum* L.) was purchased from Laiwu (Shandong, China) without disease, insect injury and mechanical damage, and stored in cold storage at −2 ± 0.5°C. Black garlic was prepared from garlic in the laboratory.

### 2.2 Isolation and purification of endophytes from garlic and black garlic

Healthy white garlic and finished black garlic were chosen, with the outermost epidermis removed. The presterilization procedure was conducted in a clean aseptic bench as follows: initial sterilization with 75% alcohol for 10 min, soak with 0.3% NaClO for 20 min, immersion with 75% alcohol for 20 min, rinse with sterile water for 20 min twice, dry for 5 min. The last flushing sterile water was spread over a petri dish containing culture medium for the beef extract peptone agar medium (BPA), potato dextrose agar medium (PDA), and Gauze's medium no. 1 (GAU). The petri dishes were incubated at 37, 28, and 28°C for 36 hr, respectively. Subsequent experiments were performed after sterile surface was confirmed (Wei et al., 2013).

The samples were inoculated using the following two methods:

1. Under aseptic conditions, samples treated with surface disinfection, including inner epidermis, clove inside, clove outside, and clove root of garlic and black garlic, were cut into 0.5 × 0.5 cm tissue block, respectively, and placed on the surface of the medium containing BPA, PDA, and GAU medium, as suggested by Cui, Pan, Zhang, Zhao, and Wei (2008). The petri dishes were incubated at 37, 28, and 28°C for 2, 5, and 2 days, respectively.
2. 25 g of garlic and black garlic samples undergoing surface disinfection were homogenized with 225 ml of sterile saline (0.9% NaCl, w/v). The mixture was stood for 15 min subsequently, which was submitted to serial 10‐fold dilutions in sterile saline to 10^−1^, 10^−2^, and 10^−3^ suspension. The 0.25 ml aliquots of diluent were spread on the surface of plates containing BPA, PDA, and GAU medium, then incubated at 37, 28, and 28°C for 2, 5, and 2 days, respectively.

After colony growth, the single colonies were picked up to three corresponding medium, and cultured at 37°C for 72 hr (Biscola et al., 2013; Wei et al., 2013). All described experiments were performed in triplicate.

### 2.3 Phenotypical characterization of endophytes from garlic and black garlic

The colonies with distinct characteristics, including morphology, size, and color, were purified using streak plate method, with an incubation at 37°C. The screening strains were transferred to a slant with solid nutrient agar medium for bacteria, or solid Gauze's medium no. 1 for actinomyces, stored at 4°C for further use (Shen, Fan, & Li, 1999). The colony characteristics visually analyzed on solid medium when cultivated to 24–48 hr. Then, the purified isolates were conducted gram staining and spore straining as described by Benson (2002), as observed under oil microscope.

The physiological and biochemical tests were conducted, respectively, as described by Buchanan & Gibbons (1984). According to results obtained, the taxonomic status of strain was acquired referring to “Bergey's Manual of Systematic Bacteriology” and “Common Bacteria Manual System Identification.”

### 2.4 Phylogenic analysis of endophytes from garlic and black garlic

Genomic DNA was extracted using TIANamp bacteria DNA kit (Qiangen, Beijing) from 3 ml of overnight culture inoculated with a single colony according to the manufacturer's instructions, used as template for amplification of the 16S rDNA fragment (Ahmadova et al., 2013).

Molecular identification was performed using 16S rDNA universal primers of bacteria 27F: (5′‐AGAGTTTGATCCTGGTCAGAACGAACGCT‐3′) and 1492R: (5′‐TACGGCTACCTTGTTACGACTTCACCCC‐3′) as described by Goto, Omura, Hara, and Sadaie (2000). The PCR system (50 μl) was composed of 2.0 μl of template DNA, 25.0 μl of 2× Taq Master Mix, 2.0 μl of 10 μmol/L forward and reverse primer each, and 19 μl of RNase‐Free Water. PCR amplification was performed under the following conditions: initial denaturation at 94°C for 3 min, 34 cycles of denaturation at 94°C for 30 s, primer annealing at 56°C for 30 s and DNA extension at 72°C for 90 s. A final extension step was added at 72°C for 10 min. The amplified products were analyzed on 1.0% (w/v) agarose gels in 5× Tris‐acetate EDTA buffer for 30 min at 100 V and made visible by UV transillumination. After the amplification products were purified, nucleotide sequencing was carried out by Sangon Biotech Co. Ltd. (Shanghai, China).

The sequences obtained were spliced and analyzed using software DNA MAN 5.0, and compared with those available in GenBank database by Basic Local Alignment Search Tool (BLAST). Then, type strains found in List of prokaryotic names with standing in nomenclature (LPSN) database and strains with high similarity were used to construct phylogenetic trees with MEGA 5.0 based on Neighbor‐Joining method (Chelo, Zé‐Zé, & Tenreiro, 2007). To determine the support for each clade, bootstrap analysis was performed with 1,000 replications.

### 2.5 Changes in total bacteria and endophytes during the black garlic processing

Fresh garlic with a simple treatment was sealed into the vacuum bag, processed in the heating oven at 80°C for 15 days as described by Zhang, Li, Lu, Liu, and Qiao (2015). Samples were immediately sealed and put back to the heating oven after daily aseptic sampling was conducted. The total number of bacteria in black garlic samples was determined by plate count agar medium (PCA) without any presterilization procedures referring to national standards of GB 4789.2–2010. The number and type of endophytes were detected using BPA medium.

The morphological characterization and 16S rDNA identification were carried as described above for garlic endophytes which were already isolated and purified. The housekeeping gene of *gyrB* gene locus was applied to confirm the strains unable to be distinguished based on 16S rDNA sequencing. PCR amplification was performed using *gyrB* universal primers UP‐1S: (5′‐GAAGTCATCATGACCGTTCTGCA‐3′) and UP‐2Sr: (5′‐AGCAGGGTACGGATGTGCGAGCC‐3′) designed according to Wang, Lee, Tai, and Kasai (2007). The PCR reaction mixture was 50 μl by reference to 16S rDNA identification. The amplification program was conducted according to La Duc, Satomi, Agata, and Venkateswaran (2004). PCR amplification products were sequenced by Sangon Biotech Co. Ltd. (Shanghai, China) after analyzed on 1% (w/v) agarose gels.

### 2.6 Partial sequencing of the *gyrA* and *rpoB* genes and multilocus sequence analysis

A set of primers, gyrA‐f (5′‐CAGTCAGGAAATGCGTACGTCCTT‐3′) and gyrA‐r (5′‐CAAGGTAATGCTCCAGGCATTGCT‐3′), corresponding to *B. subtilis gyrA* positions 43–1,070, was used to amplify *gyrA* gene. A primer pair, rpoB‐f (5′‐AGGTCAACTAGTTCAGTATGGAC‐3′) and rpoB‐r (5′‐AAGAACCGTAACCGGCAACTT‐3′), corresponding to nucleotides 6–585 of *B. subtilis rpoB* gene, was PCR amplified. The reaction mixture and PCR profile were consistent 16S rDNA sequencing, except that annealing temperature turned into 60°C for *gyrA* gene. The resultant amplicons purified were sequenced using the same primers by Sangon Biotech Co. Ltd. (Shanghai, China). The sequences of *gyrA* and *ropB* genes were aligned with reference strains using multiple‐alignment program, CLUSTALW 7.0.9. For multilocus sequence analysis, the selection of nucleotide substitution models was essential with jModelTest V2.1.4 for consensus sequences and three nucleotide fragments were combined for a congruency test using PAUP 4.0b10. Then phylogenetic inferences of the datasets were performed using the Bayesian Inference algorithm with MrBayes 3.1.2 based on best‐fitting model, following Ki, Zhang, and Qian (2009) and Weng, Chiou, Lin, and Yang (2009).

### 2.7 Effect of pH and temperature on the growth of selected strains

The effect of pH on the growth of dominant strain was determined by adjusting the pH of aliquots (30 ml) of beef extract peptone liquid medium from 3.0 up to 10.0 (with increments of one pH unit) with 1 M HCl or 1 M NaOH. After medium sterilization, the strains were incubated at 37°C for 24 hr. The OD value (λ = 600 nm) was measured every 2 hr, with blank medium as a control group, and observed the formation of bacteria membrane at the same time.

The strains preactivated for 12 hr were inoculated into flasks containing 30 ml of beef extract peptone liquid medium presterilized, which were cultivated in a thermostatic shaker incubator at 20, 30, 40, 50, and 60°C, respectively. With blank medium as a control group, the OD value (λ = 600 nm) was measured every 2 hr up to 24 hr of incubation, and observed the membrane produced at the liquid surface simultaneously.

## 3 RESULTS

### 3.1 Isolation and purification of endophytes from garlic and black garlic

As shown in Table 1, a certain number of strains were found in garlic and black garlic, whose morphological characteristics were gray wrinkled and white smooth. The number of colonies isolated from garlic was higher than that of the black garlic, indicating that some changes had taken place in microbial flora during the black garlic processing, with a slight decrease in the number. The strains grown on BPA medium were the most abundant among the three media, indicating that endophytic bacteria were dominant bacteria in garlic and black garlic. A small number of white smooth colonies were found on GAU medium, probably due to the fact that a few bacteria were grown for lack of bacteriostatic agent. After colony purification and preliminary screening, a total of 27 endophytes were found in the garlic (DS1–DS14) and black garlic (BS1–BS13). The morphological characterization and molecular biological identification were performed for screening strains.

**Table 1:** The colony growth of garlic and black garlic samples

Medium	Samples	Colony growth	Colony‐forming unit CFU/g
Potato dextrose agar medium	Garlic	Gray wrinkled colonies, White smooth colonies	140
	Black garlic	Gray wrinkled colonies, White smooth colonies	120
Beef extract peptone agar medium	Garlic	Gray wrinkled colonies, White smooth colonies	240
	Black garlic	Gray wrinkled colonies, White smooth colonies	160
Gauze's medium	Garlic	White smooth colonies	40
	Black garlic	White smooth colonies	20

### 3.2 Phenotypical characteristics of endophytes from garlic and black garlic

Morphological characteristics of the strains showed that colonies of 27 strains on solid medium incubated in aerobic conditions at 37°C for 48 hr were 2–4 mm in diameter, round, white to off‐white, smooth or rough and wrinkled, with typical characteristics of bacterial colonies. Gram staining and spore staining were conducted, indicating that 27 strains isolated from garlic and black garlic were gram‐positive *Bacillus*, with the thalli rod‐shaped or short rod‐shaped under a microscope.

Details on the physiological and biochemical characteristics of 27 isolates were shown in Table 2. All strains were able to produce protease, and most of the strains could produce extracellular amylase except DS11 and BS10 strains. All strains were capable of fermenting glucose to produce acid, and a large amount of organic acid could be produced by certain strains, which might affect the flavor of black garlic. Indole was not found for all strains. There were significant differences in the V‐P test and citrate utilization test. Based on the above results, 27 strains were identified preliminarily as *Bacillus* sp. according to “Bergey's Manual of Systematic Bacteriology.”

**Table 2:** The physiological and biochemical properties of bacteria DS1–DS14 and BS1–BS13

(a) Experiment	Result													
	DS1	DS2	DS3	DS4	DS5	DS6	DS7	DS8	DS9	DS10	DS11	DS12	DS13	DS14
Starch hydrolysis	+	+	+	+	+	+	+	+	+	+	−	+	+	+
Gelatin liquefaction	+	+	+	+	+	+	+	+	+	+	+	+	+	+
Glucose fermentation														
Acidogenic	+	+	+	+	+	+	+	+	+	+	+	+	+	+
Aerogenic	−	−	−	+	−	−	−	−	−	+	−	−	−	−
Indole test	−	−	−	−	−	−	−	−	−	−	−	−	−	−
Methyl test	−	+	−	−	−	−	−	−	+	−	−	+	−	+
V–P test	+	−	+	+	+	+	+	+	+	+	+	+	+	+
Citrate utilization	+	+	+	−	+	+	−	+	+	−	+	+	−	+

(b) Experiment	Result												
	BS1	BS2	BS3	BS4	BS5	BS6	BS7	BS8	BS9	BS10	BS11	BS12	BS13
Starch hydrolysis	+	+	+	+	+	+	+	+	+	−	+	+	+
Gelatin liquefaction	+	+	+	+	+	+	+	+	+	+	+	+	+
Glucose fermentation													
Acidogenic	+	+	+	+	+	+	+	+	+	+	+	+	+
Aerogenic	−	−	−	+	−	−	−	−	+	−	−	−	−
Indole test	−	−	−	−	−	−	−	−	−	−	−	−	−
Methyl red test	−	+	−	−	−	−	−	+	−	−	+	−	+
V–P test	−	−	+	+	+	+	+	+	+	+	+	+	+
Citrate utilization	−	+	+	−	+	+	−	+	−	+	+	−	+

+, positive; −, negative reaction.

### 3.3 Phylogenetic analysis of endophytes from garlic and black garlic

Sequences of the 16S rRNA gene are generally used as a framework for bacterial classification. In general, the similarity between sequences more than 98% could be considered as the same species (Vaishampayan et al., 2009). PCR amplification of all strains was good, with a single band at around 1,500 bp. BLAST homology analysis showed a first match with similarity above 99% with *Bacillus* for all datasets. The Neighbor‐Joining tree constructed using MEGA 5.0 indicated that 27 isolates could be categorized into eight groups. Among them, six isolates, respectively, were found to be phylogenetically related to *B. subtilis* and *B. methylotrophicus*, with 99% similarity in their 16S rDNA sequences, making *B. subtilis* sp. and *B. methylotrophicus* sp. the most dominant strains.

Considering phenotypical characteristics and phylogenetic analysis, all strains were successfully identified at the species level as shown in Table 3. There were six similar strains in garlic and black garlic, *B. aryabhattai*,* B. methylotrophicus*,* B. altitudinis*,* B. siamensis*,* B. pumilus,* and *B. subtilis*, respectively, indicating that these strains might existed in the processing of black garlic. *B. thuringiensis* was found in garlic but disappeared in black garlic, indicating that this strain could not survive in the processing of black garlic for lack of high temperature tolerance. *B*. *macroides* found in black garlic disappeared in garlic, which should be due to the microbial contamination from the environment. The above results showed that a certain number of garlic endophytes were present in garlic and black garlic, which all belonged to *Bacillus*.

**Table 3:** The phylogenic analysis results of the endophytes from garlic and black garlic

Strains	Results
DS1, DS8	*Bacillus thuringiensis* sp.
DS2, BS2	*Bacillus altitudinis* sp.
DS3, DS5, DS6, BS3, BS5, BS6	*Bacillus methylotrophicus* sp.
DS4, DS10, BS4, BS9	*Bacillus aryabhattai* sp.
DS7, DS13, BS7, BS12	*Bacillus siamensis* sp.
DS9, DS12, DS14, BS8, BS11, BS13	*Bacillus subtilis* sp.
DS11, BS10	*Bacillus pumilus* sp.
BS1	*Bacillus macroides* sp.

### 3.4 Changes in total bacteria and endophytes during the black garlic processing

As shown in Table 4, there was a certain number of garlic endophytes during the black garlic processing. Next, the number of colonies (631 ± 243 CFU/g) on PCA medium was greater than that (139 ± 54 CFU/g) of BPA medium, indicating that there were other types of strains present on the surface of garlic except for endophytes.

**Table 4:** The quantitative changes in total bacteria and endophytes during the black garlic processing

Sample no	Time (day)	Temperature (°C)	The count of endophytes (CFU/g)	The count of total bacteria (CFU/g)
A	0	80	240	1,220
B	1	80	40	140
C	2	80	180	760
D	3	80	160	800
E	4	80	180	840
F	5	80	160	640
G	6	80	180	760
H	7	80	180	800
I	8	80	200	720
J	9	80	140	580
K	10	80	120	600
L	11	80	100	520
M	12	80	100	460
N	13	80	60	360
O	14	80	100	580
P	15	80	80	320

**Figure 1:**
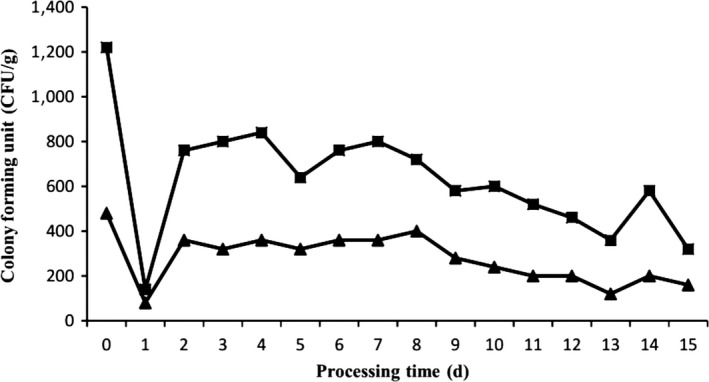
The number of total bacteria and endophytes during the black garlic processing. Fat contents: (■) total bacteria, (▲) endophytes.

The number of total bacteria and endophytes dropped sharply from an initial value (day 0) of 1,220 and 240 CFU/g to the lowest value (day 1) of 140 and 40 CFU/g during the black garlic processing (Figure 1), probably due to the lack of tolerance of soil microorganisms to high temperatures and sulfides (Avato, Tursi, Vitali, Miccolis, & Candido, 2000; Kim, Kim, & Yook, 2015). After adapting to high temperatures and sulfides, the number of total bacteria and endophytes increased rapidly due to the redifferentiation of some spores, which remained stable until the eighth day. From the ninth day, the number gradually decreased, which might be due to the increase in total phenol and total acid content and the decrease in moisture content, as well as the depletion of nutrients on the garlic surface. Throughout the process, the number of total bacteria and endophytes detected in individual days was not consistent with the overall trend. The reason might be that there were individual differences between the samples, and the microbiological culture‐dependent methods had some limitations.

### 3.5 Morphological characteristics of endophytes from the black garlic processing

Among the strains isolated and purified, a total of 78 endophytes were initially screened for further research, with eight different colony morphologies. Of these, most of the colonies were white, round, moist, smooth or off‐white, rough with irregular margins on BPA medium with the diameter from 2 to 4 mm after incubation (37°C, 24–48 hr), indicating that these forms of strains could withstand high temperature, significantly present in the black garlic processing. Microscopic analysis revealed that the majority of the isolates were endospore‐forming rods, so these isolates were expected to belong to *Bacillus* or related genera.

### 3.6 Identification of endophytes isolated from the black garlic processing

PCR amplification of all isolates was positive, with an average band length of 1,500 bp. After sequences were aligned with known sequences in the GenBank database, most strains could be identified successfully to the species level by the phylogenetic trees combined with colony morphological characteristics. However, several strains could not determine the specific species relationships due to the very high 16S rDNA/RNA gene affinity. The rate of molecular evolution inferred from *gyrB* gene sequences, a type II DNA topoisomerase, was faster than that inferred from 16S rDNA gene sequences, which could make up for the shortage of 16S rDNA gene (Wang et al., 2007). The Neighbor‐Joining tree revealed that 78 endophytes were found to be divided into 12 categories. Therefore, one strain from each category was selected for *gyrB* gene sequencing to ensure the accuracy of the identification results except for the above few strains that could not be identified by 16S rDNA sequencing.

Approximately 900 bp of the *gyrB* gene was successfully amplified for several strains according to gel electrophoresis. Trees derived from *gyrB* sequences based on GenBank database indicated that I6 belonged to *B. amyloliquefaciens*. Whereas the remaining strains belonged to *B. subtilis*. However, PCR amplification of the other strains failed despite adjusting the annealing temperature of 62, 65, 55, and 58°C. This might be due to the lack of primer specificity, which needed to redesign primers.

### 3.7 Multilocus sequence analysis

**Figure 2:**
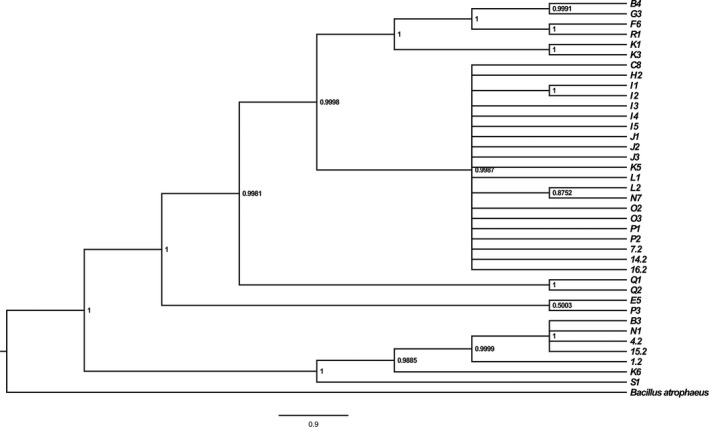
Bayesian trees inferred from the concatenated housekeeping genes (16S rDNA, *gyrA*,* ropB*), including 39 datasets determined in this study. These trees were constructed by the Bayesian method using MrBayes 3.1.2. *B. atrophaeus* was used as outgroup. The numbers at the nodes were posterior probabilities (PP).

Since isolates identified as *B. methylotrophicus*,* B. aryabhattai* and members of the *B. subtilis* group on the basis of a first identification obtained with 16S rDNA sequencing were the main garlic endophytes and their identifications were insufficient, we applied *gyrA* and *rpoB* genes to discriminate these groups of isolates and obtain more reliable species affiliation. For each of these representative strains, approximate 950‐bp *gyrA* PCR product and 550‐bp *rpoB* PCR product were generated with species‐specific primer sets. The resultant partial *gyrA* and *rpoB* sequences were assembled and aligned manually using BLAST after sequencing, indicating that the *gyrA* and *rpoB* nucleotide sequences both showed much higher variations than the 16S rDNA sequences. The sequences were compared with those from *Bacillus* reference strains available from Agricultural Research Service Culture Collection Northern Regional Research Laboratory (NRRL) and Korean Collection for Type Cultures databases. Cluster analysis inferred from *gyrA* and *rpoB* nucleotide sequences revealed that the majority of identifications were consistent with 16S rDNA sequence analysis. Only very few isolates grouped with other *Bacillus* taxa unlike previous results. The optimal model of nucleotide substitution was GTR+I+G under the Akaike Information Criterion (AIC) principle by the calculation based on jModelTest V2.1.4 for consensus sequences. Phylogeny inferred from the concatenated housekeeping genes of 39 representative strains and reference strains using the Bayesian method revealed that 39 representative strains were distributed among seven clades of the Bayesian tree (Figure 2). Among the strains, the majority of isolates indentified based on 16S rDNA sequencing were clustered together, which belonged to known type strain, *B. subtilis*. In addition, the closely related taxa of *B. subtilis* group could be discriminated phylogenetically from each other, with a significant difference over 16S rDNA identification. For example, *B. subtilis*,* B. sonorensis,* and *B. methylotrophicus* were clearly differentiated. Most of strains were distinguishable clustering with the given reference strains, in accordance with the 16S rDNA identification. However, there were several inconsistencies between the multilocus sequence analysis and previously identified results. For example, K3 strain grouped with *B. pumilus,* whereas identified as *B. aerophilus* on the basis of 16S rDNA sequencing, which highlights the advantage of *gyrA* and *rpoB* sequence analysis to supplement 16S rRNA gene sequence analysis for efficient determination of closed related species. The results of consensus identification of garlic endophytes representatives based on the 16S rDNA, *gyrA*,* gyrB,* and *rpoB* genes were shown in Table 5, as well as a portion of the 16S rDNA alone identification.

**Table 5:** Identification results of isolates from the black garlic processing

Time	Code	Strains	Time	Code	Strains
Day 0	A	*Staphylococcus epidermidis* Staphylococcus warneri Staphylococcus capitis Bacillus sonorensis Bacillus methylotrophicus *Bacillus subtilis*	Day 8	I	*Bacillus subtilis* Bacillus methylotrophicus *Bacillus amyloliquefaciens*
Day 1	B	*Staphylococcus epidermidis* Staphylococcus warneri Bacillus methylotrophicus Bacillus amyloliquefaciens Bacillus megaterium *Bacillus subtilis*	Day 9	J	*Bacillus subtilis* *Bacillus mojavensis*
Day 2	C	*Staphylococcus epidermidis* *Bacillus subtilis*	Day 10	K	*Bacillus pumilus* Bacillus altitudinis Bacillus subtilis *Bacillus methylotrophicus*
Day 3	D	*Staphylococcus epidermidis*	Day 11	L	*Bacillus subtilis*
Day 4	E	*Staphylococcus epidermidis* Staphylococcus warneri *Bacillus amyloliquefaciens*	Day 12	M	*Bacillus subtilis*
Day 5	F	*Staphylococcus epidermidis* *Bacillus megaterium*	Day 13	N	*Bacillus methylotrophicus* Bacillus amyloliquefaciens *Bacillus subtilis*
Day 6	G	*Staphylococcus epidermidis* *Bacillus subtilis*	Day 14	O	*Bacillus subtilis*
Day 7	H	*Staphylococcus epidermidis* *Bacillus subtilis*	Day 15	P	*Bacillus amyloliquefaciens* *Bacillus subtilis*

The obtained results indicated that the majority of the isolates belonged to *Bacillus* and a minority of isolates were identified as species of nonendospore‐forming genus, *Staphylococcus*. Among them, *Staphylococcus epidermidis* was present in the first 7 days of processing, and there was no detection for the strain after the seventh day, which might be due to the reduction in moisture content and the increase in phenolic compounds with the processing of black garlic. In addition to individual days, *B. subtilis* existed throughout the black garlic processing, which was the most dominant strain under culture‐dependent approaches. This strain might have a important impact on the quality of black garlic. *B. methylotrophicus* and *B. amyloliquefaciens* were also present in the black garlic processing significantly, making them the second most dominant strains. They might also affect the quality of black garlic although less than *B*. *subtilis*. The remaining strains were present in individual days of the black garlic processing, indicating that they rarely existed in the garlic, which less affected the formation of black garlic.

### 3.8 Preliminary properties of selected strains from the black garlic processing

Four strains (F7, N1, N4, N7, respectively) of *Bacillus* were selected from the garlic endophytes obtained during the black garlic processing for subsequent experiments.

As shown in Table 6, four strains could produce gelatin hydrolase and extracellular amylase. In addition, all strains were capable of fermenting glucose, lactose, sucrose, and garlic polysaccharide, with a large amount of organic acid produced, which was of importance for the flavor of black garlic. There were significant differences in the V‐P test, litmus milk test, and citrate utilization test.

**Table 6:** The physiological and biochemical properties of selected strains from the black garlic processing

Experiment	Results			
	F7	N1	N4	N7
Starch hydrolysis	+	+	+	+
Gelatin liquefaction	+	+	+	+
Glucose fermentation				
Acidogenic	+	+	+	+
Aerogenic	−	−	−	−
Lactose fermentation				
Acidogenic	+	+	+	+
Aerogenic	−	−	−	−
Sucrose fermentation				
Acidogenic	+	+	+	+
Aerogenic	−	−	−	−
Garlic polysaccharide fermentation				
Acidogenic	+	+	+	+
Aerogenic	−	−	−	−
Litmus milk test	−	+	+	+
Indole test	−	−	−	−
Methyl red test	+	+	+	+
V–P test	−	+	+	+
Citrate utilization	+	−	−	+

+, positive; −, negative reaction.

Four strains of *Bacillus* were able to grow on the medium with an initial pH of 3–10 as shown in Table 7. Specifically, the optimum growth pH of F7 strain was 5, and it showed rapid growth in wide pH range from 5 to 7 for N1 strain, which was 5–8 for N4 and N7 strains. Besides, four strains were able to grow on the medium at different temperature from 20 to 60°C and optimum growth temperature was between 30 and 40°C. No growth was established at 70°C. Notably, N1, N4, and N7 strains had a general growth at 50°C. The above results indicated that these strains could tolerate high temperature and acidic conditions, which was of great significance for playing a role during the black garlic processing.

**Table 7:** Growth of selected strains on different pH medium and different temperature

(a) Strains	pH = 3	pH = 4	pH = 5	pH = 6	pH = 7	pH = 8	pH = 9	pH = 10
F7	+	+	+++	++	++	++	++	+
N1	+	+	+++	+++	+++	++	++	+
N4	+	+	+++	+++	+++	+++	++	+
N7	+	+	+++	+++	+++	+++	++	+

(b) Strains	20°C	30°C	40°C	50°C	60°C	70°C
F7	++	+++	+++	+	+	−
N1	++	+++	+++	++	+	−
N4	++	+++	+++	++	+	−
N7	++	+++	+++	++	+	−

−, no growth; +, poor growth; ++, general growth; +++, better growth.

## 4 DISCUSSION

Black garlic was a newly processed food produced by maintaining fresh garlic at high temperature under controlled humidity condition for a long time (Liang et al., 2015), which was called fermented garlic by some researchers (Kim et al., 2012; Sato, Kohno, Hamano, & Niwano, 2006). However, there were few reports on the presence and role played by microbes during the black garlic processing. A series of studies were focused on the optimization of damp‐heat processing technology and the analysis of functional components. It was rarely related to the application of garlic endophytes as a fermenting agent to the black garlic processing (Ji et al., 2016). Therefore, the research on endophytes, which were used to accelerate the black garlic processing, enhance the black garlic flavor and functional substances and prolong the storage period, became a research hotspot.

Garlic polysaccharide, a fructans polysaccharides, was an important component of garlic as described by Wang, Huang, Zeng, and Wu (2004). During the black garlic processing, the fructans were decomposed gradually into small molecule carbohydrates, resulting in a significant increase in reducing sugar content and sweetness of black garlic (Zhang, Lei, et al., 2015). *Bacillus*, commonly found in soil, water sources and in association with plants, could withstand extreme environments and utilize a variety of carbon sources, enabling it to play a role in the processing of black garlic. It was reported that several strains, such as *B*. subtilis, *B*. aryabhattai, and *B*. coagulans had a strong capacity of acid production (Ramesh, Sharma, Sharma, Yadav, & Joshi, 2014; Wang, 2012), and the metabolites produced by certain *Bacillus* could form special flavor, such as alcohol aroma, sauce flavor and glutinous rice aroma (Cheng et al., 2014), which might have a significant impact on the flavor of black garlic. Meanwhile, some strains resembling *B*. *subtilis* and *B*. *amyloliquefaciens* could produce lipopeptide, peptide, and polyene substances, such as surfactin, iturin, and fengycin, which could inhibit the growth of pathogens (Kim et al., 2007; Raaijmakers, De Bruijn, Nybroe, & Ongena, 2010; Tan & Zou, 2001). Also, extracellular polysaccharides composed of mannose and glucose produced by several strains such as *B*. *subtilis* and *B*. *amyloliquefaciens* had a strong antioxidant activity (Yuan, Cai, Shan, Xu, & Wan, 2009). These active substances could significantly enhance the function of black garlic and extend the storage period, as well as the improvement of safety, which indicated that metabolic capabilities of *Bacillus* had important biotechnological applications. Accordingly, it was necessary to separate the endophytes in vitro and expand the culture for exploring the function of typical garlic endophytes due to their smaller number in garlic. Although there were many studies on *Bacillus* in recent years, our knowledge was incomplete and the studies remained in the initial stage. A better understanding of the identity and function of these garlic endophytes might provide information on the utilization of endophytes and the quality improvement of black garlic.

In summary, the report was the first to the isolate and identify garlic endophytes completely during the black garlic processing. The properties of dominant strains were investigated to obtain the target strains which could be applied to the black garlic processing. Our results provided theoretical evidence for the continued study of the formation mechanism of black garlic, and laid a foundation for the optimization of processing technology.

## CONFLICT OF INTEREST

The authors declare no conflict of interest.
